# Trapping of two-component light bullets in a gradient waveguide with normal group dispersion

**DOI:** 10.1371/journal.pone.0220840

**Published:** 2019-08-15

**Authors:** Aleksey A. Kalinovich, Maria V. Komissarova, Sergey V. Sazonov, Irina G. Zakharova

**Affiliations:** 1 Faculty of Physics, Lomonosov Moscow State University, Moscow, Russia; 2 National Research Centre "Kurchatov Istitute", Moscow, Russia; Universitat Politecnica de Catalunya - Campus del Baix Llobregat, SPAIN

## Abstract

In this paper we consider the process of the second harmonic generation in a gradient waveguide, taking into account diffraction and relatively weak temporal dispersion. Using the slowly varying envelope approximation and neglecting the dispersion of the nonlinear part of the response of the medium we obtain the system of parabolic equations for the envelopes of both harmonics. We also derive integrals of motion of this system. To solve it numerically we construct a nonlinear finite-difference scheme based on the Crank-Nicolson method preserving the integrals. Primarily, we focus our investigations on the processes of a two-component light bullets generation. We demonstrate that the generation of a coupled pair is possible in a planar waveguide even at normal group velocity dispersion.

## 1. Introduction

Propagation of waves in a homogeneous boundless medium is an idealization which is rarely found in nature. In most cases, the properties of the medium vary in space and this leads to a significant change in the nature of wave propagation.

Sometimes it is reasonable to add inhomogeneity to reach a desirable scenario of wave propagation. For instance, it is widely known that multi-dimensional light bullets are highly unstable in a homogeneous medium with Kerr nonlinearity. However, if we use an inhomogeneous Kerr medium, where the linear part of the refraction index depends on transverse coordinates, the wave collapse can be prevented [1–4]. The interplay between dispersion, diffraction, index inhomogeneity and Kerr nonlinearity was considered in detail in these papers. It was shown that, it is possible to form light bullets in a gradient nonlinear medium in the regime of self-defocusing and normal dispersion [3].

Light bullets at quadratic nonlinearity are more attractive than at cubic one [4]: they have a lower excitation threshold, they are more stable. Multi-component optical solitons at quadratic nonlinearity were firstly predicted in 1974 [5] when it was demonstrated that one-dimensional spatial or temporal pulse spreading could be compensated by linear self-compression. To date, the theory of multi-dimensional multi-component quadratic solitons has been essentially developed.

Spatio-temporal solitons, called also light bullets, were carefully studied either theoretically or experimentally by numerous researchers [4, 6, 7]. Recently, in [8, 9] we presented a detailed theory of breathing light bullets in a homogeneous medium with quadratic nonlinearity at anomalous dispersion. Using the averaged Lagrangian method, we derived approximate analytical solutions in the form of two-component planar spatio-temporal solitons. Besides that, an appropriate physical model was suggested for the THz range and anomalous dispersion at both frequencies.

It is known that stable multi-component optical solitons at quadratic nonlinearity are usually observed under anomalous dispersion. Including normal dispersion in consideration, one may enlarge the range of frequencies of wave localization. However, the problem of simultaneous compensation of either nonlinear or linear effects is a challenging one. By linear effects we mean diffraction and normal dispersion stretching a wave packet. As it has been already mentioned above, waveguides demonstrate a remarkable ability to support multi-dimensional soliton structures in the media with Kerr nonlinearity [1–4]. Their geometry may be chosen in such a way that it may play either focusing or defocusing role and compete with stretching tendencies. The idea to apply a similar approach to the medium with quadratic nonlinearity seems to be a promising one.

The present study of light bullets in a planar waveguide at quadratic nonlinearity is undertaken as a logical continuation of our previous investigations for homogeneous media [8, 9]. A crucial role of the competition between quadratic nonlinearity, dispersion, diffraction and inhomogeneity was discussed shortly in [10, 11].

In this paper we carefully discuss physical features of the second harmonic generation process in a gradient waveguide, taking into account diffraction and relatively weak temporal dispersion. Primarily, we focus on the process of the second harmonic generation (SHG) and the birth of a coupled pair (two-component light bullet). Using the slowly varying envelope approximation and neglecting dispersion of the nonlinear part of the medium response, we develop a governing system of equations for SHG in a waveguide with transverse inhomogeneity. We prove this system possesses two motion integrals. Thus, a numerical algorithm used for simulation must preserve difference analogs of these integrals [12,13]. Besides that, we are dealing with a multidimensional problem and, therefore, it is especially important to use algorithms that save time. Usually the splitting technique is applied to this purpose (see [12, 14, 15] and references therein). But such algorithms are not appropriate if it is necessary to preserve motion integrals of the class of problems to which the considered problem belongs [16]. Two other general approaches to the construction of numerical methods for the Schrӧdinger equations exist, namely, Fast Fourier Transform (FFT) (see also [17]) and multi-grid (MG) techniques [15, 17]. Splitting technique, FFT and MG are compared in [15]. Efficiency of MG and FFT are found close to each other. FFT methods were successfully applied to the problems of nonlinear optics [6, 18, 19]. But even FFT solvers are not sufficiently efficient in the case of 2D+1 or 3D+1 problems. Instead of them, multi-step iterative procedures could be applied for the implementation of multi-dimensional conservative difference scheme. In [20,21] a two-step iterative algorithm is proposed for a model describing the process of femtosecond optical pulse propagation in semiconductors. As the Crank-Nicolson method is used in this approach, it is conservative and is promising in efficiency which is comparable with that of the splitting technique. Thus, for the problem under consideration in our study we construct a conservative nonlinear finite-difference scheme and develop a multi-step iterative algorithm for its implementation.

The paper is organized as follows. In Sec. 2, using the quasi-optical approach, we introduce the governing equations’ system and consider in detail the way which we utilize for the description of transversal inhomogeneity. In Sec. 3 we describe the numerical algorithm used to perform direct numerical simulation and discuss its properties. Results of direct numerical simulation are discussed in Sec. 4. Sec. 5 contains the conclusions.

## 2. Equation system of SHG in a waveguide

### 2.1. Quasi-optical approach

The way to derive the equation describing the interplay between dispersion, diffraction, inhomogeneity, and nonlinearity was described in detail in [3] for Kerr nonlinearity. There the authors started from Maxwell’s equations supplemented by the dependence of the refractive index on carrier frequency and transversal coordinates. Then a standard procedure was fulfilled, the paraxial and the slowly varying envelope approximations were applied. It is important to underline that the equation for a slowly varying envelope obtained in that paper is similar to the standard multidimensional nonlinear Schrodinger equation (NLSE) except the only one additional term due to nonlinear medium inhomogeneity.

In the present work for the consideration of the SHG in a quadratically nonlinear waveguide we applied about the same procedure.

To describe waveguide geometry we represent the linear frequency susceptibility *χ*_*ω*_(**r**_⊥_) in the form

$$\chi_{\omega }(r_{⊥})=\chi_{\omega }^{\left(0\right)}\left[1+f_{\omega }(r_{⊥})\right].$$

Here **r**_⊥_ is the transverse radius-vector perpendicular to the central axis of the waveguide, $\chi_{\omega }^{\left(0\right)}$ is the linear susceptibility of the medium at the center of the waveguide cross section; the dimensionless function *f*_*ω*_(**r**_⊥_) characterizes the transverse inhomogeneity of the susceptibility and satisfies the condition *f*_*ω*_(0) = 0.

Similar to [3], the paraxial approximation is also applied. We assume that both harmonics propagate along the *z*-axis, and at that, we suppose the linear group velocities $v_{g}^{\left(\omega \right)}$ and $v_{g}^{\left(2\omega \right)}$ (envelope carrier frequencies *ω* and 2*ω* correspondingly) at the center of the waveguide (**r**_⊥_ = 0) are related by the following inequality

$$\left|v_{g}^{\left(2\omega \right)}-v_{g}^{\left(\omega \right)}\right|<<v_{g}^{\left(2\omega \right)},v_{g}^{\left(\omega \right)}.$$

Provided inhomogeneity, nonlinearity, dispersion, and diffraction are weak, we use the slowly varying envelope approximation [22]. The resulting system of equations for the envelopes of the fundamental Φ_1_ and second Φ_2_ harmonics looks as follows:

$$\begin{array}{l} i\left(\frac{\partial \Phi_{1}}{\partial z}+\delta \frac{\partial \Phi_{1}}{\partial \tau }\right)=\omega g_{1}(r_{⊥})\Phi_{1}-\frac{\beta_{\omega }}{2}\frac{\partial^{2}\Phi_{1}}{\partial \tau^{2}}+\alpha_{\omega }\Phi_{1}^{*}\Phi_{2}e^{i(2k_{1}-k_{2})z}+\frac{c}{2\omega n_{\omega }^{\left(0\right)}}\Delta_{⊥}\Phi_{1},\\ i\left(\frac{\partial \Phi_{2}}{\partial z}-\delta \frac{\partial \Phi_{2}}{\partial \tau }\right)=2\omega g_{2}(r_{⊥})\Phi_{2}-\frac{\beta_{2\omega }}{2}\frac{\partial^{2}\Phi_{2}}{\partial \tau^{2}}+\alpha_{2\omega }\Phi_{1}^{2}e^{-i(2k_{1}-k_{2})z}+\frac{c}{4\omega n_{2\omega }^{\left(0\right)}}\Delta_{⊥}\Phi_{2}. \end{array}$$ (1.1)

In (1.1) $\tau =t-\frac{1}{2}\left(\frac{1}{v_{g}^{\left(\omega \right)}}+\frac{1}{v_{g}^{\left(2\omega \right)}}\right)$, *t* is time,

$$g_{1}(r_{⊥})=\frac{n_{\omega }^{(0)2}-1}{2cn_{\omega }^{\left(0\right)}}f_{\omega }(r_{⊥}),\;g_{2}(r_{⊥})=\frac{n_{2\omega }^{(0)2}-1}{2cn_{2\omega }^{\left(0\right)}}f_{2\omega }(r_{⊥}).$$ (1.2)

*f*_*ω*_(**r**_⊥_) and *f*_2*ω*_(**r**_⊥_) are the dimensionless functions satisfying the condition *f*_*ω*_(0) = *f*_2*ω*_(0) = 0, $\delta =\frac{1}{2}\left(\frac{1}{v_{g}^{\left(\omega \right)}}-\frac{1}{v_{g}^{\left(2\omega \right)}}\right)$ is the mismatch of group velocities, *k*_1_ = *k*(*ω*) and *k*_2_ = *k*(2*ω*) are the wave numbers, corresponding to the fundamental frequency *ω* and second harmonics 2*ω*, respectively. *β*_*ω*_ and *β*_2*ω*_ are the parameters of the dispersion of group velocities (DGV), $n_{\omega }^{\left(0\right)}$ and $n_{2\omega }^{\left(0\right)}$ are the refractive indexes at the center of the waveguide cross section of the fundamental and second harmonics, respectively, *c* is the speed of light in vacuum, Δ_⊥_ is the transversal Laplacian, $\alpha_{\omega }=\frac{2\pi \omega }{cn_{\omega }^{\left(0\right)}}\chi^{\left(2\right)}(2\omega ,-\omega )$, $\alpha_{2\omega }=\frac{4\pi \omega }{cn_{2\omega }^{\left(0\right)}}\chi^{\left(2\right)}(\omega ,\omega )$. *χ*^(2)^(2*ω*,−*ω*) and *χ*^(2)^(*ω*,*ω*) are the second order nonlinear optical susceptibilities at the waveguide center.

The first and second terms in the right-hand sides of Eq (1.1) describe the effect of the waveguide (transverse inhomogeneity) on the phase and group velocities of the harmonics, respectively. Spatial inhomogeneity of the linear refractive indices for both harmonics in (1.1) is taken into account in the same way as it was done in [3, 4] when studying the waveguide propagation mode of the soliton of the NLSE.

Putting in (1.1) *g*_1,2_ = 0, we come to the well-known system of equations for the pulsed mode of SHG in a homogeneous medium [23,24].

Dependences of the refractive indexes *n*_*ω*,2*ω*_ on the transverse coordinates **r**_⊥_ of the waveguide are expressed through the functions *f*_*ω*,2*ω*_(**r**_⊥_) as follows:

$$n_{\omega ,2\omega }^{2}(r_{⊥})=1+\left(n_{\omega ,2\omega }^{(0)2}-1\right)\left(1+f_{\omega ,2\omega }(r_{⊥})\right).$$ (1.3)

If we deal with a focusing waveguide, then the functions *f*_*ω*_(**r**_⊥_) and *f*_2*ω*_(**r**_⊥_) decrease from the center to the periphery. In the opposite case, the waveguide is defocusing. Below we consider planar waveguides, i.e. Δ_⊥_ = ∂^2^/∂*x*^2^ and *f*_*ω*,2*ω*_ = *f*_*ω*,2*ω*_(*x*), the profiles of the waveguide functions are conveniently chosen, for example, in the form of parabolic profile with saturation:

$$f_{\omega ,2\omega }(x)=\varepsilon_{w}\frac{x^{2}}{a_{\omega ,2\omega }^{2}+x^{2}}.$$ (1.4)

Here *x* is the distance from the centre of the waveguide to a current point in transversal direction, *ε*_*w*_ = 1 for the defocusing waveguide and *ε*_*w*_ = −1 for the focusing waveguide.

In addition, we can consider profiles in the forms

$$f_{\omega ,2\omega }(x)=\varepsilon_{w}\tanh^{2}\left(\frac{x}{a_{\omega ,2\omega }}\right)$$ (1.5)

or

$$f_{\omega ,2\omega }(x)=\varepsilon_{w}\left[1-\exp\left(-\frac{x^{2}}{a_{\omega ,2\omega }^{2}}\right)\right].$$ (1.6)

Note that in the case of focusing waveguides (*ε*_*w*_ = −1) the transverse susceptibility profiles (see (1.3)) for the dependences (1.4), (1.5) and (1.6) have the Lorentzian, exponential and Gaussian modes, respectively:

$$n_{\omega ,2\omega }^{2}(x)=1+\left(n_{\omega ,2\omega }^{(0)2}-1\right)\frac{a_{\omega ,2\omega }^{2}}{a_{\omega ,2\omega }^{2}+x^{2}},$$ (1.7)

$$n_{\omega ,2\omega }^{2}(x)=1+\left(n_{\omega ,2\omega }^{(0)2}-1\right){\mathrm{sech}}^{2}\left(\frac{x}{a_{\omega ,2\omega }}\right),$$ (1.8)

$$n_{\omega ,2\omega }^{2}(x)=1+\left(n_{\omega ,2\omega }^{(0)2}-1\right)\exp\left(-\frac{x^{2}}{a_{\omega ,2\omega }^{2}}\right).$$ (1.9)

In all these cases, the refractive indexes *n*_*ω*,2*ω*_(*x*) decrease from the maximum value at the center of the waveguide (at *x* = 0) to unity at its periphery (at *x*→∞).

### 2.2. Dimensionless equations and integrals of motion

We still consider a planar (Δ_⊥_ = ∂^2^/∂*x*^2^) waveguide and use dimensionless parameters related to the physical parameters in the following way: Φ_1,2_ = *A*_1,2_*A*_*in*_, $z=\bar{z}l_{nl}$, $x=\bar{x}R_{in}$, $\tau =\bar{\tau }\tau_{in}$, $\Delta \bar{k}=\Delta kl_{nl}$, Δ*k* = 2*k*_1_−*k*_2_, *l*_*nl*_ = (*α*_*ω*_*A*_*in*_)^−1^, $a_{\omega ,2\omega }=R_{in}{\bar{a}}_{\omega ,2\omega }$. Here *A*_*in*_ is the input peak amplitude of the fundamental harmonic, *R*_*in*_ and *τ*_*in*_ are initial pulse spatial and temporal widths, respectively. We introduce also the following propagation and waveguide characteristics: $D_{q1}=\frac{2\pi \omega l_{nl}}{cn_{\omega }^{\left(0\right)}{\bar{a}}_{\omega }^{2}}\chi_{\omega }^{\left(0\right)}$, $D_{q2}=\frac{4\pi \omega l_{nl}}{cn_{2\omega }^{\left(0\right)}{\bar{a}}_{2\omega }^{2}}\chi_{2\omega }^{\left(0\right)}$, $D_{\tau 1}=\frac{\beta_{\omega }l_{nl}}{2\tau_{in}^{2}}$, $D_{\tau 2}=\frac{\beta_{2\omega }l_{nl}}{2\tau_{in}^{2}}$, $D_{x1}=\frac{cl_{nl}}{2\omega n_{\omega }^{\left(0\right)}R_{in}^{2}}$, $D_{x2}=\frac{cl_{nl}}{4\omega n_{2\omega }^{\left(0\right)}R_{in}^{2}}$, *γ* = *α*_2*ω*_/*α*_*w*_, *a*_*w*_ and *a*_2*ω*_ are the characteristic lengths of waveguide transversal inhomogeneity. Then, we suppose the group velocity matching conditions are satisfied ($v_{g}^{\left(\omega \right)}=v_{g}^{\left(2\omega \right)}=v_{g}$). Finally, we get the following system of the dimensionless equations which we use as a base for our numerical simulation:

$$i\frac{\partial A_{1}}{\partial \bar{z}}=D_{q1}p_{1}(\bar{x})A_{1}-D_{\tau 1}\frac{\partial^{2}A_{1}}{\partial {\bar{\tau }}^{2}}+A_{1}^{*}A_{2}e^{i\Delta \bar{k}\bar{z}}+D_{x1}\frac{\partial^{2}A_{1}}{\partial {\bar{x}}^{2}},i\frac{\partial A_{2}}{\partial \bar{z}}=D_{q2}p_{2}(\bar{x})A_{2}-D_{\tau 2}\frac{\partial^{2}A_{2}}{\partial {\bar{\tau }}^{2}}+\gamma A_{1}^{2}e^{-i\Delta \bar{k}\bar{z}}+D_{x2}\frac{\partial^{2}A_{2}}{\partial {\bar{x}}^{2}},0<z<L_{z},(\bar{x},\bar{\tau })\in \Gamma ,\Gamma =\left\{-L_{x}/2<\bar{x}<L_{x}/2\right\}\times \left\{-L_{\tau }/2<\bar{\tau }<L_{\tau }/2\right\}.$$ (2.1)

Boundary and initial conditions are as follows.

$$A_{1,2}(\bar{z},-L_{x}/2,\bar{\tau })=A_{1,2}(\bar{z},L_{x}/2,\bar{\tau })=A_{1,2}(\bar{z},\bar{x},-L_{\tau }/2)=A_{1,2}(\bar{z},\bar{x},L_{\tau }/2)=0,A_{1}(0,\bar{x},\bar{\tau })=A_{10}(\bar{x},\bar{\tau }),A_{2}(0,\bar{x},\bar{\tau })=A_{20}(\bar{x},\bar{\tau }).$$ (2.2)

In (2.1)–(2.2) *L*_z_ is the dimensionless length of the nonlinear medium, *L*_*τ*_ is the dimensionless time interval during which laser pulse interaction with the medium is analyzed. *L*_*x*_ is the dimensionless length of the transversal domain. We choose finite lengths of the transversal coordinate and time with zero conditions at the boundaries of these coordinates from the following considerations. Since we deal with a finite pulse, it is naturally to take a sufficiently long time interval whose boundaries are not influenced by the pulse. Besides, the transversal size of the studied bullet is also finite due to narrow laser radiation. Thus, we can choose a wide transversal domain with boundaries which are not affected by radiation. Obviously, this choice assumes the need to monitor the fulfillment of zero boundary conditions, and, if necessary, a widening of the computational domain. It may cause specific computational difficulties, which nevertheless, can be eliminated by imposing artificial boundary conditions (see [25] and references therein).

In (2.1) *p*_1,2_ are dimensionless functions describing waveguide inhomogeneity and corresponding to (1.4)–(1.6). Below we discuss simulation with

$$p_{1,2}=\frac{{\bar{x}}^{2}}{{\bar{a}}_{\omega ,2\omega }^{2}},$$ (2.3)

$$p_{1,2}=\frac{{\bar{x}}^{2}}{1+{\bar{x}}^{2}/{\bar{a}}_{\omega ,2\omega }^{2}},$$ (2.4)

$$p_{1,2}={\bar{a}}_{\omega ,2\omega }^{-2}\tanh^{2}\left(\frac{\bar{x}}{{\bar{a}}_{\omega ,2\omega }^{}}\right),$$ (2.5)

$$p_{1,2}={\bar{a}}_{\omega ,2\omega }^{-2}\left[1-\exp\left(-\frac{{\bar{x}}_{}^{2}}{{\bar{a}}_{\omega ,2\omega }^{2}}\right)\right].$$ (2.6)

Here $\bar{x}$ is the distance from the center of the waveguide to the current point. In general, *a*_*ω*_ and *a*_2*ω*_ are not equal to each other ($a_{\omega ,2\omega }={\bar{a}}_{\omega ,2\omega }R_{in}$). Form (2.3) describes a waveguide with parabolic profile, (2.4)–(2.5) correspond to profiles with Lorentzian (2.4), tangential (2.5), and exponential (2.6) saturation. Below we will omit bars in the notations of dimensionless variables.

The system (2.1)–(2.2) possesses the motion integrals

$$I_{1}=\overset{\infty }{\underset{-\infty }{\iint dxd\tau }}\left(\gamma {\left|A_{1}\right|}^{2}+{\left|A_{2}\right|}^{2}\right),$$ (2.7)

$$\begin{array}{l} I_{3}=\overset{\infty }{\underset{-\infty }{\iint dx}}d\tau \{-2\gamma \left|A_{1}^{2}A_{2}\right|\cos(2\varphi_{1}-\varphi_{2})+\Delta k{\left|A_{2}\right|}^{2}+2\gamma D_{x1}{\left|\frac{\partial A_{1}}{\partial x}\right|}^{2}+D_{x2}{\left|\frac{\partial A_{2}}{\partial x}\right|}^{2}-\\ -2\gamma D_{\tau 1}{\left|\frac{\partial A_{1}}{\partial \tau }\right|}^{2}-D_{\tau 2}{\left|\frac{\partial A_{2}}{\partial \tau }\right|}^{2}-2\gamma D_{q1}p_{1}(x){\left|A_{1}\right|}^{2}-D_{q2}p_{2}(x){\left|A_{2}\right|}^{2}\} \end{array}$$ (2.8)

In (2.8) *φ*_1,2_ are the wave phases. To derive (2.7) we multiply the equations of (2.1) by $\gamma A_{1}^{*},A_{2}^{*}$ correspondingly, then integrate both parts of these transformed equations with respect to *x*,*τ*, sum them up and take real parts of all components. Finally we get the equality (2.7) expressing a law of energy conservation. At the next step we proceed, multiplying the equations of (2.1) by $2\gamma \frac{\partial A_{1}^{*}}{\partial z},\frac{\partial A_{2}^{*}}{\partial z}$ correspondingly. Then we repeat the successive steps used when receiving (2.7), but at the last step we take imaginary parts of all components. So, we obtain (2.8) concerning the evolution of the phases of harmonics.

## 3. Numerical approach

### 3.1. Approximation of the equations

We introduce uniform grids in the domain Γ and in the *z* domain:

$$\begin{array}{l} \omega_{\Gamma }=\omega_{x}\times \omega_{\tau }=\left\{(x_{j},\tau_{k})=(jh_{x}-L_{x}/2,kh_{\tau }-L_{\tau }/2);j=1,2,\ldots ,N_{x}-1;k=1,2,\ldots ,N_{\tau }-1;h_{x}=L_{x}/N_{x};h_{\tau }=L_{\tau }/N_{\tau }\right\},\\ \omega_{z}==\left\{z_{l}=lh_{z};l=1,2,\ldots ,N_{z}-1,h_{z}=L_{z}/N_{z}\right\}. \end{array}$$ (3.1)

A numerical approximation to the exact solution of the problem (2.1)–(2.2) $A_{1,2}^{l,j,k}=A_{1,2}(z_{l},x_{j},\tau_{k})$ we consider on the grid *ω*_*z*_×*ω*_Γ_ and denote it by $\Psi_{1,2}^{l,j,k}=\Psi_{1,2}(z_{l},x_{j},\tau_{k})$. To approximate first- and second-order derivatives with respect to *x* and *τ* we use the standard expressions: $\Psi_{1,2}^{l,j,k}{}_{\bar{\tau }\tau }=\frac{\Psi_{1,2}^{l,j,k+1}-2\Psi_{1,2}^{l,j,k}+\Psi_{1,2}^{l,j,k-1}}{h_{\tau }^{2}}$ and $\Psi_{1,2}^{l,j,k}{}_{\bar{x}x}=\frac{\Psi_{1,2}^{l,j+1,k}-2\Psi_{1,2}^{l,j,k}+\Psi_{1,2}^{l,j-1,k}}{h_{x}^{2}}$ are used for $\frac{\partial^{2}A_{1,2}}{\partial \tau^{2}}$ and $\frac{\partial^{2}A_{1,2}}{\partial x^{2}}$. We also introduce the notation for the half-sums: $\overset{0.5}{\Psi_{1,2}^{l,j,k}}=\left(\Psi_{1,2}^{l+1,j,k}+\Psi_{1,2}^{l,j,k}\right)/2$. Then we write down the following nonlinear symmetric finite difference scheme in the case of phase matching (Δ*k* = 0):

$$\begin{array}{l} \frac{\Psi_{1}^{l+1,j,k}-\Psi_{1}^{l,j,k}}{h_{z}}-iD_{\tau 1}{\overset{0.5}{\Psi_{1_{\bar{\tau }\tau }}}}^{l,j,k}+i\overset{0.5}{{\left(\Psi_{1}^{l,j,k}\right)}^{*}}\overset{0.5}{\Psi_{2}^{l,j,k}}=\\ -iD_{q1}p_{1}(x_{j}^{}){\overset{0.5}{\Psi_{1}}}^{l,j,k}-iD_{x1}{\overset{0.5}{\Psi_{1_{\bar{x}x}}}}^{l,j,k},\\ \frac{\Psi_{2}^{l+1,j,k}-\Psi_{2}^{l,j,k}}{h_{z}}-i{\overset{0.5}{D_{\tau 2}\Psi_{2_{\bar{\tau }\tau }}}}^{l,j,k}+i\overset{0.5}{\gamma {\left(\Psi_{1}^{l,j,k}\right)}^{2}}=\\ -iD_{q2}p_{2}(x_{j}^{}){\overset{0.5}{\Psi_{2}}}^{l,j,k}-iD_{x2}{\overset{0.5}{\Psi_{2_{\bar{x}x}}}}^{l,j,k}. \end{array}$$ (3.2)

Initial and boundary conditions are approximated exactly.

$$\Psi_{1,2}(z_{l},-L_{x}/2,\tau_{k})=\Psi_{1,2}(z_{l},L_{x}/2,\tau_{k})=\Psi_{1,2}(z_{l},x_{j},-L_{\tau }/2)=\Psi_{1,2}(z_{l},x_{j},L_{\tau }/2)=0,\Psi_{1}(0,x_{j},\tau_{k})=\Psi_{10}(x_{j},\tau_{k}),\Psi_{2}(0,x_{j},\tau_{k})=\Psi_{20}(x_{j},\tau_{k}).$$ (3.3)

This scheme is known to be of the second order of approximation with respect to all coordinates [12–14]. It is easily generalized to the case of phase mismatch (Δ*k*≠0).

### 3.2. Two-step iterative process

Nonlinear scheme (3.2)–(3.3) can be resolved with the help of an iteration process [12]. But the computational complexity of the direct matrix inversion after linearization makes such an approach practically useless [12, 15]. FFT technique [15, 19] is not straightforward in the case under consideration due to the *x*-dependent coefficients *p*_1,2_ in (3.2). Therefore, we develop the approach proposed in [20, 21] and write down the following two-step iteration process for the implementation of (3.2)- (3.3).

At the first step we seek for the iteration (s+1) of the difference functions ${\overset{s+1}{\Psi }}_{1,2}^{l+1,j,k}$:

$$\begin{array}{l} \frac{{\overset{s+1}{\Psi_{1}}}^{l+1,j,k}-\Psi_{1}^{l,j,k}}{h_{z}}-iD_{\tau 1}{\overset{\overset{S+1}{0.5}}{\Psi_{1_{\bar{\tau }\tau }}}}^{l,j,k}+i\overset{\overset{S}{0.5}}{{\left(\Psi_{1}^{l,j,k}\right)}^{*}}\overset{\overset{S}{0.5}}{\Psi_{2}^{l,j,k}}=\\ -iD_{q1}p_{1}(x_{j}^{}){\overset{\overset{S}{0.5}}{\Psi_{1}}}^{l,j,k}-iD_{x1}{\overset{\overset{S}{0.5}}{\Psi_{1_{\bar{x}x}}}}^{l,j,k}, \end{array}$$ (3.4)

$$\begin{array}{c} \frac{{\overset{s+1}{\Psi }}_{2}{}^{l+1,j,k}-\Psi_{2}^{l,j,k}}{h_{z}}-iD_{\tau 1}{\overset{\overset{s+1}{0.5}}{\Psi_{2_{\bar{\tau }\tau }}}}^{l,j,k}+i\gamma \overset{\overset{s}{0.5}}{{\left(\Psi_{1}^{l,j,k}\right)}^{2}}=-iD_{q2}p_{2}(x_{j}^{}){\overset{\overset{s}{0.5}}{\Psi_{2}}}^{l,j,k}-iD_{x2}{\overset{\overset{s}{0.5}}{\Psi_{2_{\bar{x}x}}}}^{l,j,k}.\\ {\overset{s+1}{\Psi }}_{1,2}(z_{l},-L_{x}/2,\tau_{k})={\overset{s+1}{\Psi }}_{1,2}(z_{l},L_{x}/2,\tau_{k})={\overset{s+1}{\Psi }}_{1,2}(z_{l},x_{j},-L_{\tau }/2)={\overset{s+1}{\Psi }}_{1,2}(z_{l},x_{j},L_{\tau }/2)=0,\\ {\overset{s=0}{\Psi }}_{1,2}^{l+1,j,k}=\Psi_{2}^{l,j,k}. \end{array}$$ (3.5)

At the second step we complete the procedure at the current iteration cycle finding ${\overset{s+2}{\Psi }}_{1,2}^{l+1,j,k}$:

$$\begin{array}{l} \frac{{\overset{s+2}{\Psi_{1}}}^{l+1,j,k}-\Psi_{1}^{l,j,k}}{h_{z}}-iD_{\tau 1}{\overset{\overset{S+1}{0.5}}{\Psi_{1_{\bar{\tau }\tau }}}}^{l,j,k}+i\overset{\overset{S+1}{0.5}}{{\left(\Psi_{1}^{l,j,k}\right)}^{*}}\overset{\overset{S+1}{0.5}}{\Psi_{2}^{l,j,k}}=\\ -iD_{q1}p_{1}(x_{j}^{}){\overset{\overset{S+1}{0.5}}{\Psi_{1}}}^{l,j,k}-iD_{x1}{\overset{\overset{S+2}{0.5}}{\Psi_{1_{\bar{x}x}}}}^{l,j,k}, \end{array}$$ (3.6)

$$\begin{array}{c} \frac{{\overset{s+2}{\Psi }}_{2}{}^{l+1,j,k}-\Psi_{2}^{l,j,k}}{h_{z}}-iD_{\tau 2}{\overset{\overset{s+1}{0.5}}{\Psi_{2_{\bar{\tau }\tau }}}}^{l,j,k}+i\gamma \overset{\overset{s+1}{0.5}}{{\left(\Psi_{1}^{l,j,k}\right)}^{2}}=-iD_{q2}p_{2}(x_{j}){\overset{\overset{s+1}{0.5}}{\Psi_{2}}}^{l,j,k}-iD_{x2}{\overset{\overset{s+2}{0.5}}{\Psi_{2_{\bar{x}x}}}}^{l,j,k}.\\ {\overset{s+2}{\Psi }}_{1,2}(z_{l},-L_{x}/2,\tau_{k})={\overset{s+2}{\Psi }}_{1,2}(z_{l},L_{x}/2,\tau_{k})={\overset{s+2}{\Psi }}_{1,2}(z_{l},x_{j},-L_{\tau }/2)={\overset{s+2}{\Psi }}_{1,2}(z_{l},x_{j},L_{\tau }/2)=0. \end{array}$$ (3.7)

We see that at each step of the iteration process we deal with one-dimensional problem. Thus, matrix inversion in (3.4)–(3.7) can be made with the help of the tridiagonal matrix algorithm, and in general, the proposed method is time-saving. The iteration procedure stops when the criterion $\underset{0\leq j\leq N_{x}^{},0\leq k\leq N_{\tau }}{\max}\left|{\overset{s+2}{\Psi }}_{1,2}{}^{l+1,j,k}-{\overset{s}{\Psi }}_{1,2}{}^{l+1,j,k}\right|\leq \varepsilon ,$ where *ε* is a constant determining computation accuracy.

**Theorem 3.1.** *Provided that h*_*z*_≤*C(h*_*x*_*h*_*τ*_*)*^2^ *the unique solution of the difference scheme (3*.*2)-(3*.*3) exists and the two-step iteration process (3*.*4)-(3*.*7) converges to it as a geometric progression with the denominator q*≈*h*_*z*_/(*h*_*x*_*h*_*τ*_)^2^.

**Proof.** The proof is made with the help of the contraction mapping theorem. We have to demonstrate that (3.4)–(3.7) is a contraction, i.e that all iterations are uniformly limited and that in a certain difference norm ‖∙‖_(*h*)_

$${\left\|{\overset{s+2}{\Psi }}_{1,2}{}^{l+1,j,k}-{\overset{s+1}{\Psi }}_{1,2}{}^{l+1,j,k}\right\|}_{\left(h\right)}\leq q{\left\|{\overset{s+1}{\Psi }}_{1,2}{}^{l+1,j,k}-{\overset{s}{\Psi }}_{1,2}{}^{l+1,j,k}\right\|}_{\left(h\right)}.$$ (3.8)

Passing to the difference norm C

$${\left\|{\overset{s}{\Psi }}_{1,2}{}^{l+1}\right\|}_{C(h)}=\underset{0\leq j\leq N_{x},0\leq k\leq N_{\tau }}{\max}\left|{\overset{s}{\Psi }}_{1,2}{}^{l+1,j,k}\right|,$$

we use at each step of the iteration process the Cauchy–Bunyakovsky–Schwarz inequality, estimate the inverse norm of the difference operators $F_{\bar{x}x}$ and $F_{\bar{\tau }\tau }$ [12]. This procedure allows us firstly, to show that the iterations are uniformly limited if *h*_*z*_≤*C(h*_*x*_*h*_*τ*_*)*^2^. Then, considering consecutively the iteration differences ${\left\|{\overset{s+1}{\Psi }}_{1,2}{}^{l+1}-{\overset{s}{\Psi }}_{1,2}{}^{l+1,}\right\|}_{C(h)}$, ${\left\|{\overset{s+2}{\Psi }}_{1,2}{}^{l+1}-{\overset{s+1}{\Psi }}_{1,2}{}^{l+1,}\right\|}_{C(h)}$, and using similar estimations, we get the inequality (3.8).

### 3.3. Conservativeness

**Theorem 3.2.** *The scheme (3*.*1)-(3*.*2) is conservative*: *it preserves the following difference analogues of the integrals (2*.*7)-(2*.*8) in the norm*

$$\begin{array}{c} {\left\|F\right\|}_{L^{2}(h)}^{2}={\left(F,F\right)}_{\left(h\right)},{\left(F,G\right)}_{\left(h\right)}=\sum_{j=0}^{N_{x}}\sum_{k=0}^{N_{\tau }}F^{j,k}{\left(G^{j,k}\right)}^{*}h_{x}h_{\tau }:\\ I_{1(h)}=\gamma {\left\|\Psi_{1}^{l+1}\right\|}_{L^{2}(h)}^{2}+{\left\|\Psi_{2}^{l+1}\right\|}_{L^{2}(h)}^{2}, \end{array}$$ (3.9)

$$\begin{array}{l} I_{3}=-2\gamma {\left\|\left(\Psi_{1}^{l+1}\right)\sqrt{\left|\Psi_{2}^{l+1}\cos\left(2\arg\left(\Psi_{1}^{l+1}\right)-\arg\left(\Psi_{2}^{l+1}\right)\right)\right|}\right\|}_{L^{2}(h)}^{}+\Delta k{\left\|\Psi_{2}^{l+1}\right\|}_{L^{2}(h)}^{2}+2\gamma D_{x1}{\left\|\Psi_{1}^{l+1}{}_{\overset{}{x}}\right\|}_{L^{2}(h)}^{2}+\\ +D_{x2}{\left\|\Psi_{2}^{l+1}{}_{\overset{}{x}}\right\|}_{L^{2}(h)}^{2}-2\gamma D_{\tau 1}{\left\|\Psi_{1}^{l+1}{}_{\overset{}{\tau }}\right\|}_{L^{2}(h)}^{2}-D_{\tau 2}{\left\|\Psi_{2}^{l+1}{}_{\overset{}{\tau }}\right\|}_{L^{2}(h)}^{2}-2\gamma D_{q1}p_{1}^{j}{\left\|\Psi_{1}^{l+1}\right\|}_{L^{2}(h)}^{2}-D_{q2}p_{2}^{j}{\left\|\Psi_{2}^{l+1}\right\|}_{L^{2}(h)}^{2} \end{array}$$ (3.10)

In (3.10) $\Psi_{1,2_{\tau }}^{l+1}=\frac{\Psi_{1,2_{}}^{l+1,j,k+1}-\Psi_{1,2_{}}^{l+1,j,k-1}}{2h_{\tau }}$, $\Psi_{1,2_{\tau }}^{l+1}=\frac{\Psi_{1,2_{}}^{l+1,j+1,k}-\Psi_{1,2_{}}^{l+1,j-1,k}}{2h_{x}}$.

**Proof.** We follow the procedure described in [13]. Multiplying both parts of the first equation of (3.1) by $\overset{0.5}{(\gamma \Psi_{1}^{l,j,k}}{)}^{*}h_{x}h_{\tau }$ and both parts of the second equation by $\overset{0.5}{(\Psi_{2}^{l,j,k}}{)}^{*}h_{x}h_{\tau }$ and summing them up with respect to *x* and *τ*, we take the real parts of the resulting expressions. Then we apply the difference analogue of the integration by parts formula [12] and finally, get (3.8).

We use a similar procedure for the integral (2.3). But to derive (3.9) we multiply both equations of (3.1) by $\frac{{\left(\gamma \Psi_{1}^{l+1,j,k}\right)}^{*}-{\left(\gamma \Psi_{1}^{l,j,k}\right)}^{*}}{h_{z}}$, $\frac{{\left(\Psi_{2}^{l+1,j,k}\right)}^{*}-{\left(\Psi_{2}^{l,j,k}\right)}^{*}}{h_{z}}$, sum them up with respect to *x*, τ and take the imagine parts of the obtained equations.

## 4. Numerical simulation in a planar waveguide and discussion

### 4.1. Initial pulse and absorbing boundary conditions

In this Section we discuss the outcome of a direct numerical simulation of the system (2.1) on the base of the finite-difference scheme (3.2). In computations we launch the initial pulse at both frequencies

$$A_{1}^{}(z=0)=\exp\left(-x^{2}-\tau^{2}\right),\;A_{2}(z=0)=\frac{1}{2}\exp\left(-x^{2}-\tau^{2}\right)$$ (4.1)

or at the fundamental frequency only

$$A_{1}^{}(z=0)=\exp\left(-x^{2}-\tau^{2}\right),\;A_{2}(z=0)=0.$$ (4.2)

Implementing calculations we usually choose *L*_*x*_ and *L*_*τ*_ so that the amplitudes of both harmonics decay to zero at the boundaries of this domain. In particular cases, when *L*_*x*_ and *L*_*τ*_ are too large, the efficiency of the method can be improved by using absorbing boundary conditions along the coordinates *x* and *τ*. To this end we embed an artificial absorption in the system (2.1)

$$i\frac{\partial A_{1}}{\partial z}=D_{q1}p_{1}(x)A_{1}-i\sigma (x,\tau )A_{1}-D_{\tau 1}\frac{\partial^{2}A_{1}}{\partial \tau^{2}}+A_{1}^{*}A_{2}e^{i\Delta kz}+D_{x1}\frac{\partial^{2}A_{1}}{\partial x^{2}},$$ (4.3)

$$i\frac{\partial A_{2}}{\partial z}=D_{q2}p_{2}(x)A_{2}-i\sigma (x,\tau )A_{2}-D_{\tau 2}\frac{\partial^{2}A_{2}}{\partial \tau^{2}}+\gamma A_{1}^{2}e^{-i\Delta kz}+D_{x2}\frac{\partial^{2}A_{2}}{\partial x^{2}},$$ (4.4)

defining the function *σ*(*x*,*τ*) = *σ*_min_(*σ*_*x*_(*x*)+*σ*_*τ*_(*τ*)), where

$$\sigma_{x}(x)=\{\begin{array}{c} 0,\;-L_{x}/2+x_{ab}<x<L_{x}/2-x_{ab}\\ \exp(-(-L_{x}/2+x_{ab}-x)\varepsilon_{x}),\;x<-L_{x}/2+x_{ab}\\ \exp(-(L_{x}/2-x_{ab}+x)\varepsilon_{x}),\;x>L_{x}/2-x_{ab} \end{array},$$

$$\sigma_{\tau }(\tau )=\{\begin{array}{c} 0,\;-L_{\tau }/2+\tau_{ab}<\tau <L_{\tau }/2-\tau_{ab}\\ \exp(-(-L_{\tau }/2+\tau_{ab}-\tau )\varepsilon_{\tau }),\;\tau <-L_{\tau }/2+\tau_{ab}\\ \exp(-(L_{\tau }/2-\tau_{ab}+\tau )\varepsilon_{\tau }),\;\tau >L_{\tau }/2-\tau_{ab} \end{array}.$$

**Fig 1:**
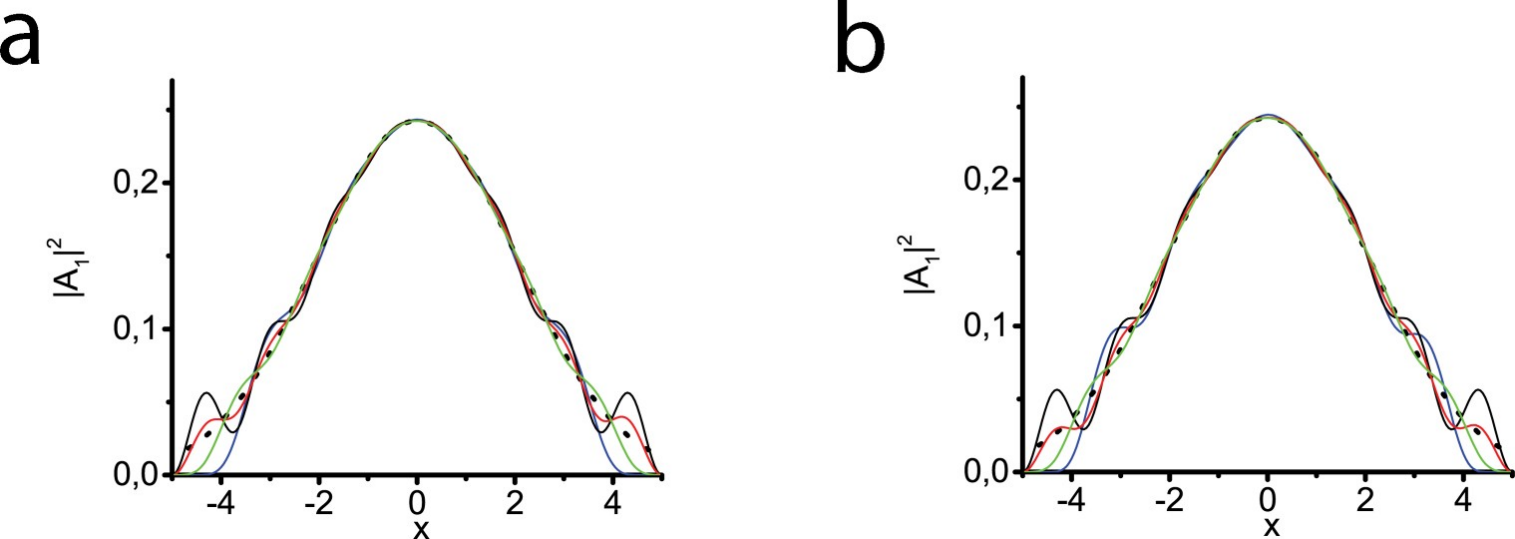
Linear diffraction of the Gaussian beam of the initial unit radius at the distance z = 10. *x*_*ab*_ = 0.2, *L*_*x*_ = 2. Solid black lines in figures (a,b) represent diffraction with zero boundary conditions at the points *x* = −5 and *x* = 5, at that, *σ*_min_ = 0. Dotted black lines correspond to computations with transversal calculation area which is much larger than that shown in the figures (a,b), *σ*_min_ = 0 (reference curves). Beam profile dependence on *σ*_min_ for *ε*_*xτ*_ = 5 (a). *σ*_min_ = 0.01 (red line), *σ*_min_ = 0.1 (green line), *σ*_min_ = 1 (blue line). Beam profile dependence on *ε*_*xτ*_ for *σ*_min_ = 0.01 (b). *ε*_*xτ*_ = 2 (red line), *ε*_*xτ*_ = 5 (green line), *ε*_*xτ*_ = 7 (blue line).

Fig 1 illustrates our choice of the function *σ* and the values of the constants included in it as well. We demonstrate the selection of parameters for *σ*_*x*_(*x*). Our considerations concerning *σ*_*τ*_(*τ*) are similar. In our computations we take the values *x*_*ab*_≈(0.1÷0.2)*L*_*x*_, *τ*_*ab*_≈(0.1÷0.2)*L*_*τ*_ and the constants *σ*_min_≈0.01÷0.1, *ε*_*xτ*_≈3÷5. Analyzing Fig 1(A), which represents linear diffraction of the pulse, one can see that changing the value of *σ*_min_ at fixed values of *x*_*ab*_, *L*_*x*_, and *ε*_*xτ*_, we observe the best coincidence with the reference curve obtained with the larger computational transversal domain (black dotted line) at *σ*_min_ = 0.1 (green line). Fig 1(B) demonstrates the choice of optimal *ε*_*xτ*_ = 5 in a similar way. Here we fix *x*_*ab*_, *L*_*x*_, and *σ*_min_ and vary *ε*_*xτ*_. Parameter *x*_*ab*_ allows us to improve absorption but reduces the useful part of the computational domain. Values of *τ*_*ab*_ are taken from the considerations of computational efficiency and minimization of the tails’ influence on the bullet formation process. Thus, we can reduce the computational domain along the transverse coordinates. It is obvious that in such calculations the integrals (2.7)–(2.8) may not be conserved. Typically we take the domains *L*_*x*_ = 20, *L*_*τ*_ = 30 which are longer than those presented in Fig 1. It seems these dimensions are rather redundant. In fact, in the process of bullet trapping the pulse-beam spreads in time and *x* and parts of it which are close to the boundaries of absorption domains may be of intensities comparable with the peak ones. Chosen dimensions allow us to minimize this effect. At the same time they are shorter by an order of magnitude than those that should be taken in the absence of absorption layers.

Our computations are carried out on the basis of the difference scheme described in the Sec. 3. We choose also the following computational parameters: the step size along the propagation coordinate is *h*_*z*_ = 0.001, while the step sizes along space and time coordinates are *h*_*x*_ = 0.05, *h*_*τ*_ = 0.05. Typical size of the computational domain along z axis in our simulation is *L*_*z*_ = 500.

### 4.2. Simulation of light bullet trapping in different regimes

Since anomalous dispersion supports light bullets in 2D+1 media even without waveguide [6, 8], our principal interest is to investigate waveguide at normal group velocity dispersion. The simulation and observation are conducted up to *z* = 500*l*_*nl*_. Such distance is an optimal one to investigate effects appearing due to the interference of diffraction, dispersion, nonlinearity and waveguide geometry.

In the first series of numerical experiments, we use a pulse of the Gaussian form at both frequencies (4.1) as a two-component input radiation. This case implies the phase-matching conditions *k*_2_ = 2*k*_1_ or Δ*k* = 0. We have demonstrated that the regime of robust soliton propagation is established with the termination of energy exchange approximately at 200*l*_*nl*_. This propagation is accompanied by regular in-phase oscillations of peak intensities at both harmonics. The generalized phase Φ = 2*φ*_1_−*φ*_2_−Δ*kz* oscillates near optimal value equal to −*π*. It also confirms the formation of a stable parametrically coupled pair of solitons and means that we observe the reactive regime of the classic parametric solitons when there is no energy exchange between the fundamental and second harmonics [5]. We have examined the temporal and spatial profiles at *z* = 500*l*_*nl*_ as well. Due to waveguide influence the spatial profile is narrower than the temporal one. The calculated profiles have been compared with those of the Gaussian form having the same amplitudes, widths, and durations. This comparison demonstrates quite a good match between them. Thus, the soliton solution in this case has the form close to the Gaussian one. In total, in this computational series we have showed the trapping of a two-component “breathing” light bullet and its stability. One should underline that it forms at normal dispersion due to waveguide geometry only.

**Fig 2:**
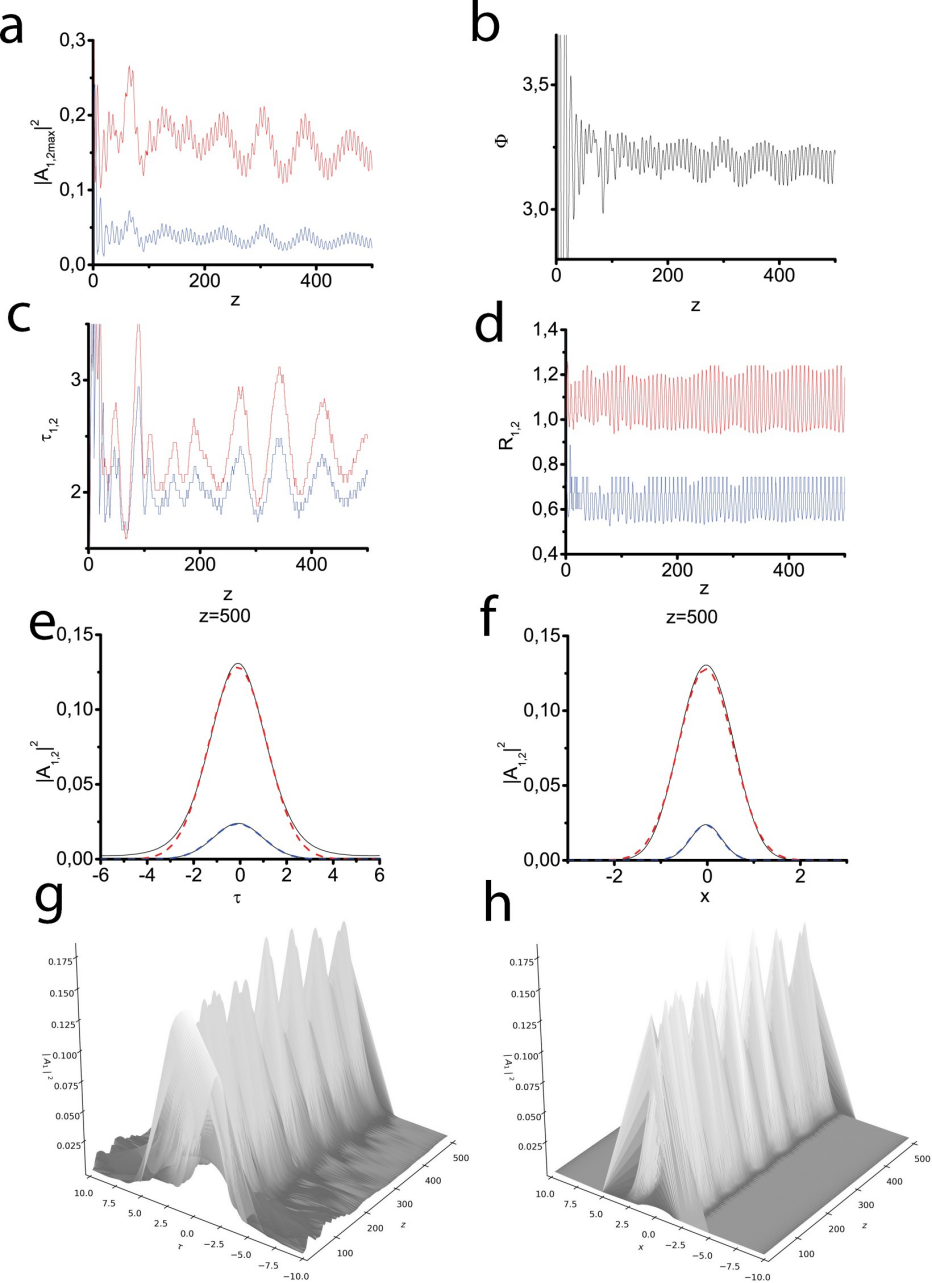
Light bullet formation in the process of SHG at normal dispersion in a focusing waveguide. Input pulse of the Gaussian form (4.2) is launched at the fundamental frequency only. Peak intensities of the fundamental (red line) and second (blue line) harmonics vs the propagation distance (a). Generalized phase vs the propagation distance (b). Temporal width of the fundamental (red line) and second (blue line) harmonics vs the propagation distance (c). Spatial width of the fundamental (red line) and second (blue line) harmonics vs the propagation distance (d). Transversal profiles of both harmonics calculated at the distance z = 500 (black solid lines) (e), (f), the approximations of the Gaussian form (4.1) having the same amplitudes, durations (e) and spatial widths (f) (red and blue dashed lines for the fundamental and second harmonics, correspondingly). Temporal and spatial profile evolution for the fundamental harmonic (g), (h). Waveguide with parabolic profile (2.4). Parameters: *D*_*q*1_ = −0.5, *D*_*q*2_ = −1.0, *D*_*x*1_ = 0.1, *D*_*x*2_ = 0.05, *D*_*τ*1_ = 0.1, *D*_*τ*2_ = 0.2, Δ*k* = 0, *γ* = 0.5.

Consecutive processes of SHG and two-component light bullet formation are in the focus of the second series of our numerical simulation. In this experiment we launch a pulse in the Gaussian form (4.1) at the fundamental frequency only. Fig 2(A) demonstrates the dependence of the peak intensities of both harmonics on the propagation coordinate *z*. Firstly, energy transfer and second harmonic generation are observed. Approximately at a distance of 100*l*_*nl*_ we see that both waves are trapped in a coupled pair and the regime of robust two-component soliton propagation is established. As in the previous case wave propagation is accompanied by regular in-phase oscillations of peak intensities at both harmonics (Fig 2(A)). Peak amplitude maxima are lower than in the previous case due to the lower initial total energy. Fig 2(B) shows the generalized phase oscillations near optimal value equal to *π*. In contrast to the previous case, the optimal generalized phase changes a sign. Note that the generalized phase is calculated as *arccos* function in the following way. If at a current point it is close to 2*π*, then at the next point the value 2*πm* is added to the calculated value in order to avoid a jump. Thus, at long distances the phase will be approximately 2*πm* (*m*>1 if it increases, or *m*<−1, if it decreases). Then, when a soliton is trapped, the phase will be close to *π*+2*πm*. The dimensionless temporal (Fig 2(C)) and spatial (Fig 2(D)) widths of the harmonics oscillates in-phase to each other and in-anti-phase to the intensity. Due to the influence of waveguide geometry spatial profile (Fig 2(F)) is narrower than a temporal one (Fig 2(E)). Fig 2(G) and Fig 2(H) show the evolution of temporal and spatial intensity profiles, correspondingly, at the fundamental frequency along the longitudinal coordinate *z*.

**Fig 3:**
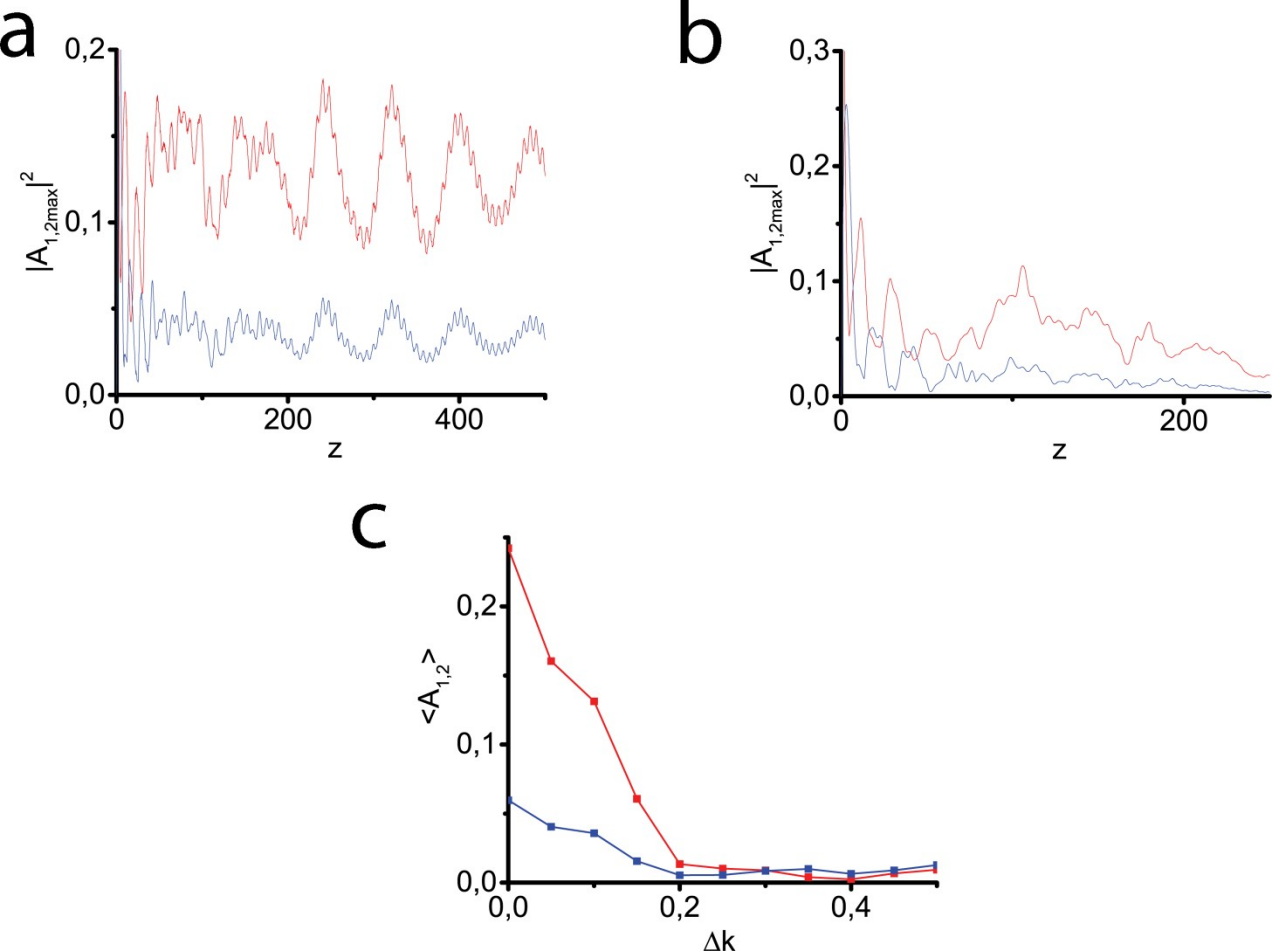
Light bullet formation in the process of SHG at the phase mismatch Δ*k* = 0.1 (a) and the Gaussian pulse evolution at Δ*k* = 0.15 (b). In (a,b) red lines depict the fundamental harmonic intensities, the blue lines correspond to those of the second harmonic. Dependence of averaged amplitudes of the first (red line) and second (blue line) harmonics on phase mismatch. Maximum amplitudes (in time and transversal coordinate) of the first (red line) and second (blue line) harmonics, averaged over the distance from *z* = 100 to *z* = 200 (c). Input pulse of the Gaussian form (4.2) is launched at the fundamental frequency only. Other parameters are the same as in Fig 2.

Fig 3 illustrates the consecutive second harmonic generation and two-component light bullet trapping provided a small phase-mismatching. We see that the process of light bullet propagation is rather stable at Δ*k* = 0.1 (Fig 3(A)). If we increase the value of the phase velocity detuning up to 0.5, it distorts the picture of the formation of a bullet due to a sufficient decay of the resulting pulse intensity. Even at Δ*k* = 0.15 we observe a soliton close to the stability limit (Fig 3(B)). High values of phase mismatching prevent SHG. Fig 3(C) represents maximum amplitudes of both harmonics in time and transversal coordinate, which are averaged over the distance from *z* = 100 to *z* = 200. It can be seen that, starting from Δ*k* = 0.2, the averaged maximum amplitudes drastically fall and soliton formation does not occur.

**Fig 4:**
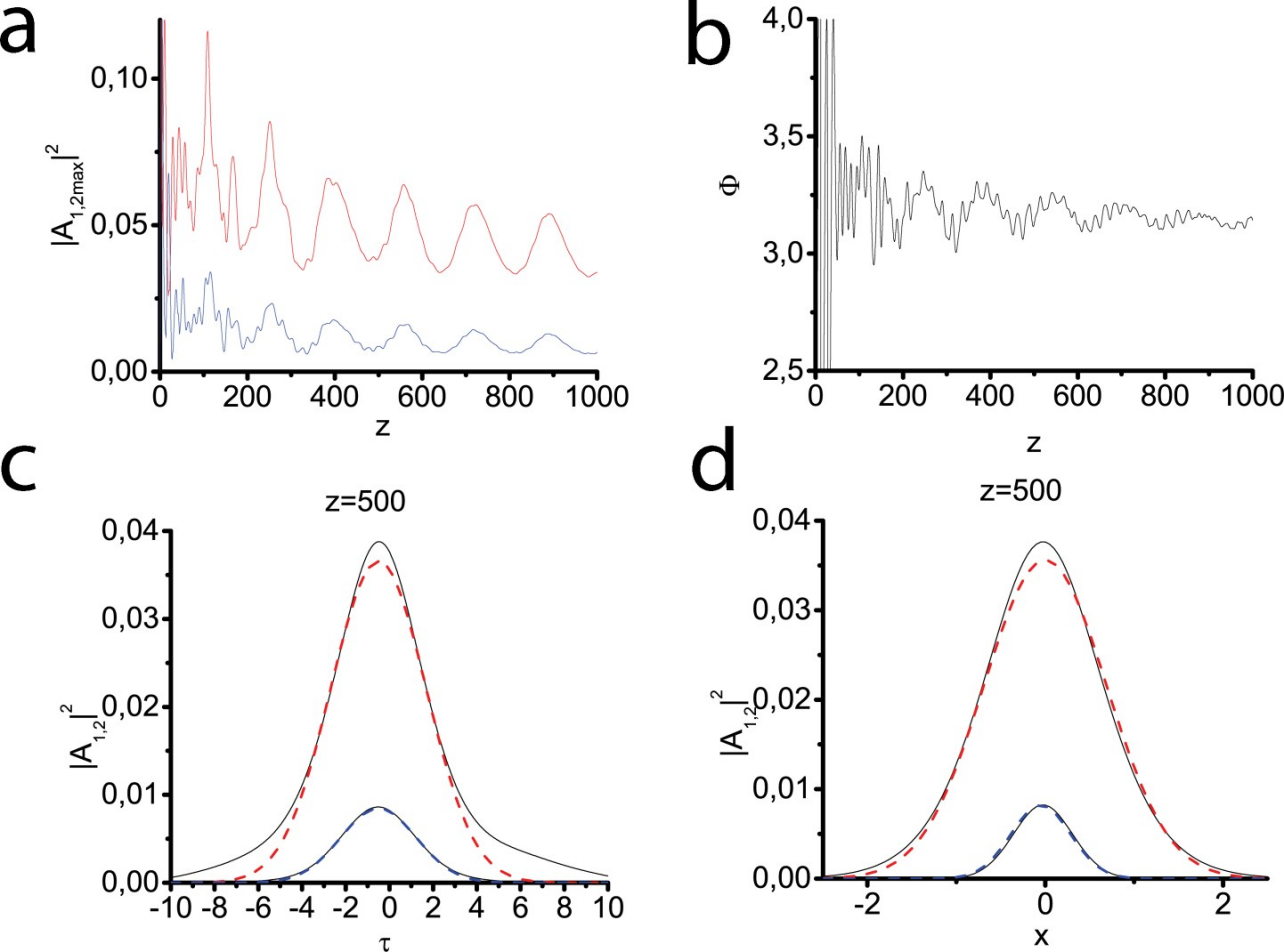
Peak intensities of the fundamental (red line) and second (blue line) harmonics vs the propagation distance (a). Generalized phase vs the propagation distance (b). Transversal profiles of both harmonics calculated at the distance z = 500 (black solid lines) (c), (d), the approximations of the form (4.1) with the maximum intensity and temporal (c) and spatial (d) widths of the calculated first and second harmonics (red blue dashed lines). The Gaussian input pulse at the fundamental frequency (4.2) is launched into the waveguide with the Lorentzian profile (2.4). Parameters: *D*_*q*1_ = −0.5, *D*_*q*2_ = −1.0, *D*_*x*1_ = 0.1, *D*_*x*2_ = 0.05, *D*_*τ*1_ = 0.1, *D*_*τ*2_ = 0.2, Δ*k* = 0, *γ* = 0.5.

**Fig 5:**
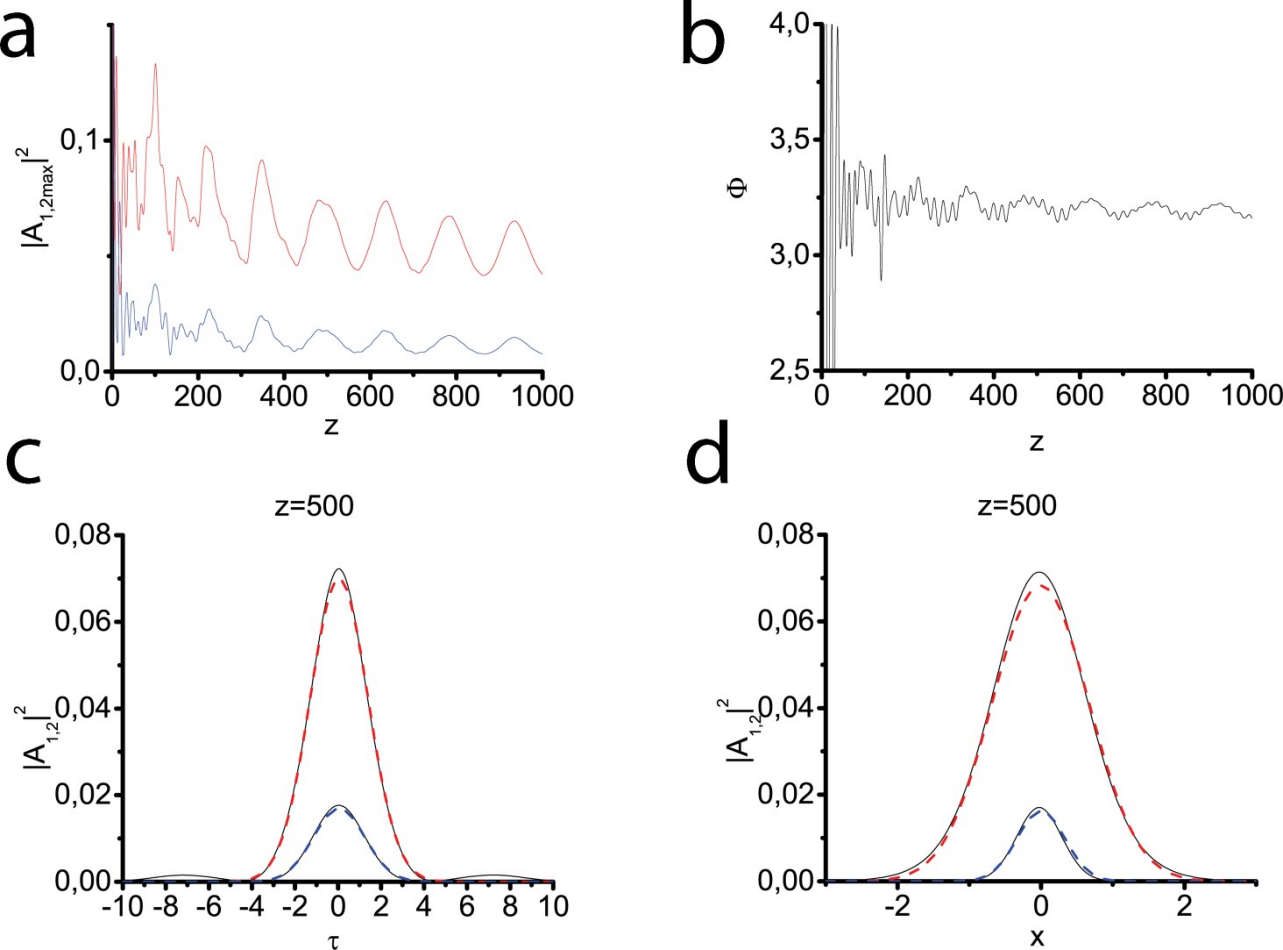
The same as in Fig 4 but waveguide has a tangential profile (2.5).

**Fig 6:**
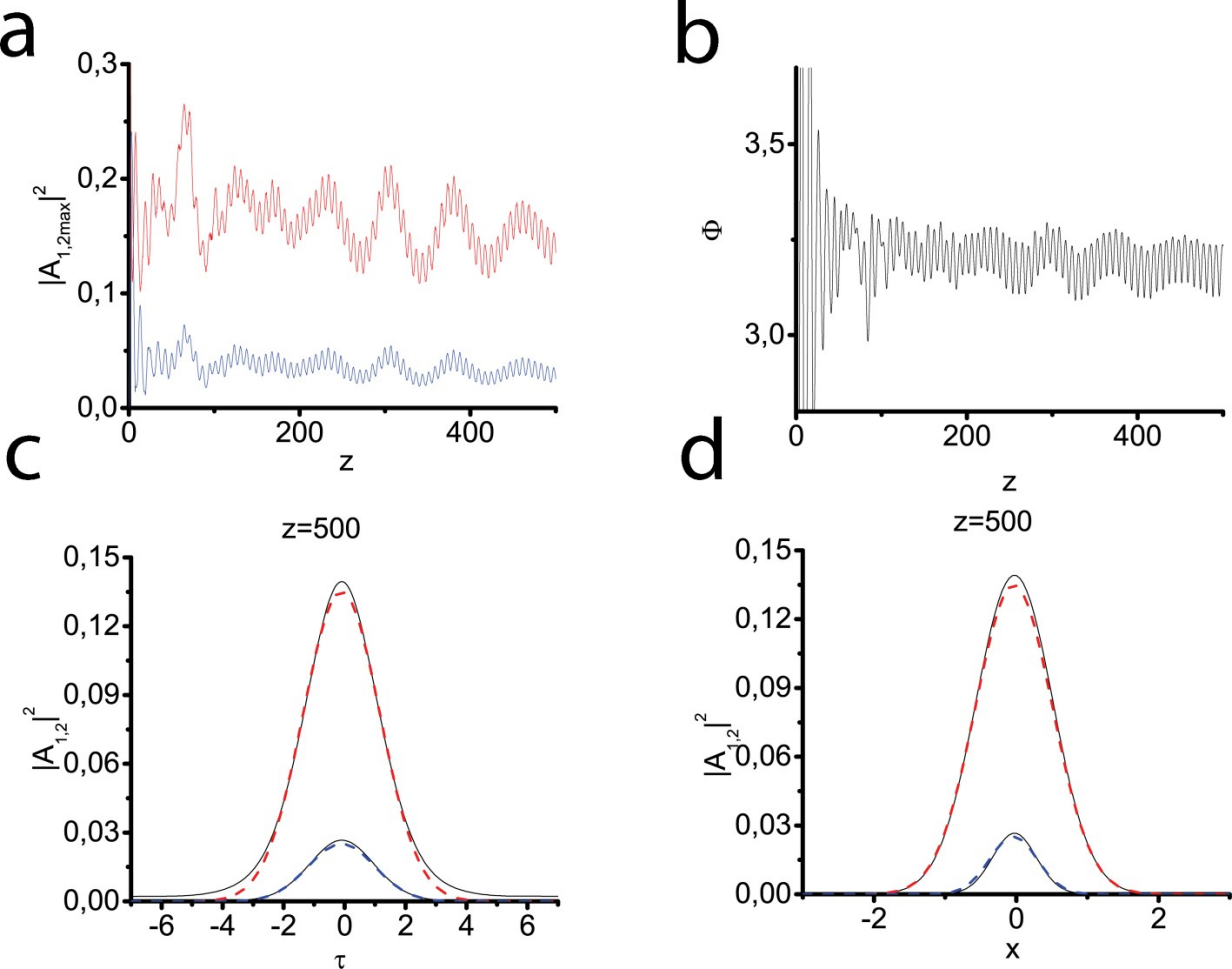
The same as in Fig 4 but waveguide has the Gaussian profile (2.6).

As it was discussed in the Sec. 2.1 the transverse susceptibility profiles may have different form. The next series of experiments deals with the Lorentzian (Fig 4), tangential (Fig 5) and Gaussian (Fig 6) modes, respectively. The consecutive SHG and two-component light bullet formation is demonstrated in all three cases. The most evident confirmation of this fact can be received when we analyze the behavior of generalized phase–at the distance of 50*l*_*nl*_ it begins to oscillate near optimal value equal to *π*. Since the waveguide in these cases effectively influences only on pulse-beams of a finite width, the soliton tails can gradually move away from the pulse center, a part of the pulse energy gradually goes to the periphery. This results in forming a quasi-stable bullet little by little losing its energy. In Fig 4(C) and Fig 5(C) it can be seen that the time profile also strongly deviates from the Gaussian one. At the same time, the generalized phase experiences significant oscillations, which indicate an obvious deviation of this regime from the soliton one. For the Gaussian profile (Fig 6), the deviations from the basic test case (Fig 2) are less noticeable. It may be explained by a good match between initial beam size and waveguide size.

**Fig 7:**
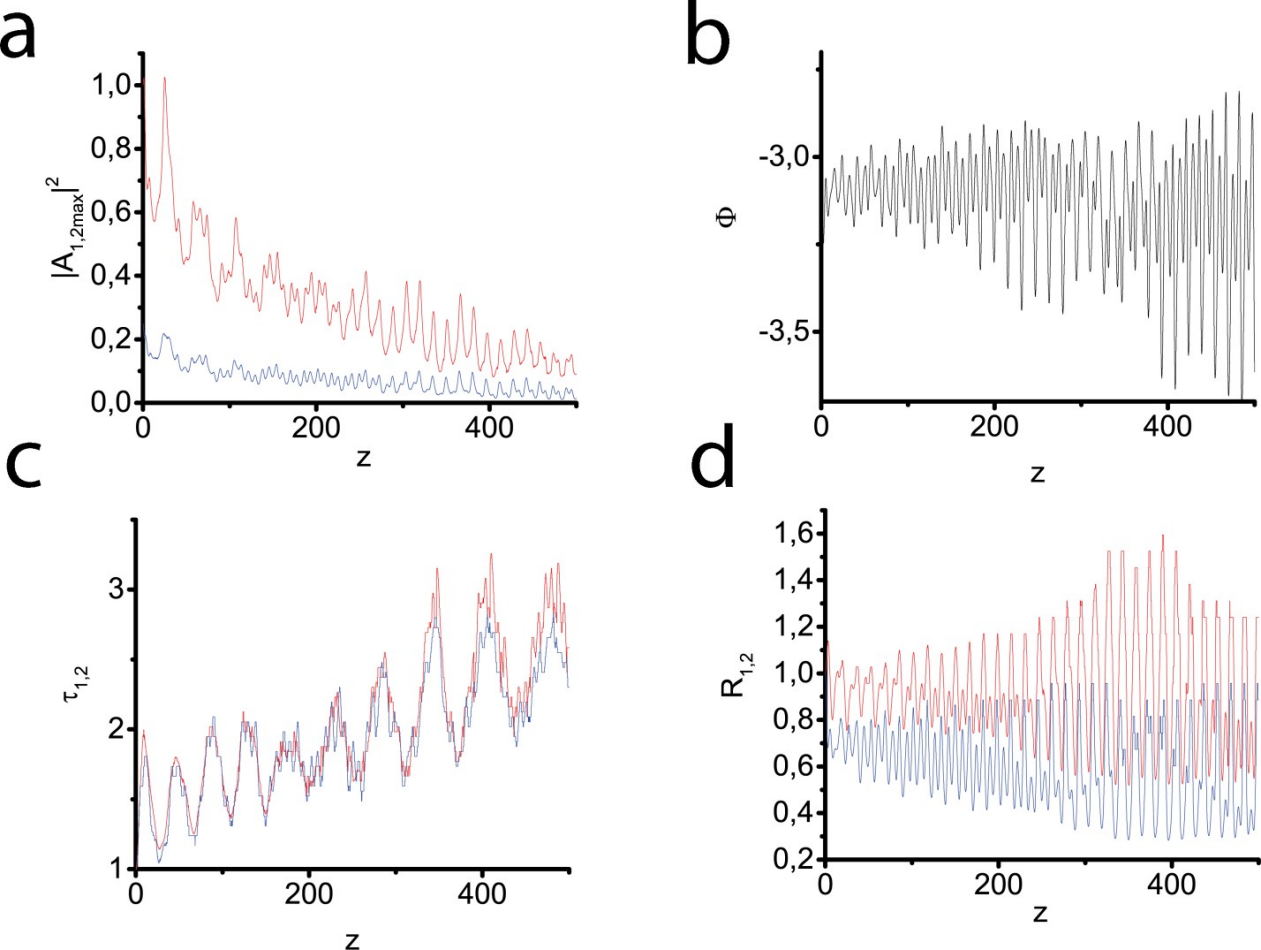
Peak intensities of the fundamental (red line) and second (blue line) harmonics vs the propagation distance (a). Generalized phase vs the propagation distance (b). Temporal width of the fundamental (red line) and second (blue line) harmonics vs the propagation distance (c). Spatial width of the fundamental (red line) and second (blue line) harmonics vs the propagation distance (d). Input pulses of the Gaussian form at both frequencies (4.1), waveguide with parabolic profile (2.3) is at the fundamental frequency only. Parameters: *D*_*q*1_ = −0.5, *D*_*q*2_ = 0, *D*_*x*1_ = 0.025, *D*_*x*2_ = 0.0125, *D*_*τ*1_ = 0.1, *D*_*τ*2_ = 0.2, Δ*k* = 0, *γ* = 0.5.

Fig 7 illustrates a remarkable property of the investigated system to capture both pulses in a waveguide and to trap a two-component light bullet while initially the waveguide presents at one frequency only. In this experiment we launch input pulses of the Gaussian form at both frequencies (4.1), provided waveguide with parabolic profile (2.3) is just at the fundamental frequency. We see that the bullet energy gradually decreases down to bullet disappearing. However, one should note that the distance of total bullet destruction is not so small–it is equal approximately to 30 dispersion lengths. It is worthwhile to underline once again that provided normal dispersion, trapping of a two-component light bullet is impossible in the absence of a waveguide at least at the fundamental frequency.

## 5. Conclusions

To trap multi-component light bullets at normal dispersion of group velocities one should balance nonlinearity, dispersion, and diffraction. It is a challenging problem requiring the presence of an additional “power” compressing the pulses. Waveguides may play such a positive role, therefore, it was reasonable to investigate in detail a possibility of spatial-temporal solitons formation and propagation in a waveguide with quadratic nonlinearity.

Using the quasi-optical approach, we introduce a system of equations describing the propagation of pulse-beams in gradient waveguides. Numerical method for direct system simulation in the planar case is developed. A distinctive feature of the method is the preservation of the integrals of motion which are intrinsic to the governing system. Numerical simulation has been performed for various sets of parameters. Since light bullets at anomalous dispersion form and propagate even without waveguide, our principal interest was to investigate waveguide at normal group velocity dispersion. The formation of optical bullets has been shown when launching the input Gaussian pulse at both frequencies and at the fundamental frequency only as well. Besides that, we have computed the cases of phase mismatch and various types of waveguide profiles, in which soliton solutions also trapped. If a waveguide is only at the fundamental frequency, the formation of soliton-like solutions manifests itself too but its energy is gradually decreased. Nevertheless, as a whole it spreads to tens of dispersion lengths. In general, the simulations performed show a possibility of the existence of optical bullets and quasi-soliton solutions in the presence of normal dispersion in quadratically nonlinear waveguides under various conditions.
